# FOXA1 in Breast Cancer: A Luminal Marker with Promising Prognostic and Predictive Impact

**DOI:** 10.3390/cancers14194699

**Published:** 2022-09-27

**Authors:** Jasna Metovic, Fulvio Borella, Marta D’Alonzo, Nicoletta Biglia, Luca Mangherini, Cristian Tampieri, Luca Bertero, Paola Cassoni, Isabella Castellano

**Affiliations:** 1 Department of Oncology, University of Turin, 10126 Turin, Italy; 2 Division of Gynecology and Obstetrics 1, City of Health and Science University Hospital, University of Turin, 10126 Turin, Italy; 3 Department of Surgical Sciences, University of Turin, 10126 Turin, Italy; 4 Academic Division of Obstetrics and Gynaecology, A.O. Ordine Mauriziano, University of Turin, 10128 Turin, Italy; 5 Department of Medical Sciences, University of Turin, 10126 Turin, Italy

**Keywords:** FOXA1, breast cancer, estrogen receptor, androgen receptor, prognostic marker, predictive marker

## Abstract

**Simple Summary:**

Forkhead box A1 protein (FOXA1) is described as a pioneer factor that binds to condensed chromatin, permitting the recruitment of other transcription factors to the DNA. Worthy of note, FOXA1 is an interacting partner of both the estrogen and androgen receptor, playing a crucial role in the development and progression of breast cancer. Moreover, it is necessary for the estrogen-receptor-binding and subsequent transcriptional activation of luminal genes in breast cancer cells. Herein, we review principal roles of FOXA1 in normal and neoplastic tissues, with special attention to its prognostic and predictive role in luminal and non-luminal breast cancers.

**Abstract:**

The present review focuses on the function of the forkhead protein FOXA1 in breast cancer (BC) in relation to steroid hormone receptors. We explored the currently available analytic approaches for FOXA1 assessment both at gene and protein levels, comparing the differences between the available techniques used for its diagnostic assessment. In addition, we elaborated on data regarding the prognostic and predictive role of this marker in BC based on several studies that evaluated its expression in relation to the outcome and/or response to therapy. FOXA1, similar to the androgen receptor (AR), may have a dual role in BC according to hormonal status. In luminal cancers, its expression contributes to a better prognosis, while in triple-negative breast cancers (TNBC), it implies an adverse outcome. Consequently, we observed that FOXA1-positive expression in a neoadjuvant setting may predict a lack of response in luminal BC as opposed to TNBC, in which FOXA1 allegedly increases its chemosensitivity. In conclusion, considering its accessible and convenient identification by immunohistochemistry, its important impact on prognosis, and its suitability to identify patients with different responses to chemotherapy, we propose that FOXA1 could be tested in routine diagnostics as an additional prognostic and predictive marker in BC.

## 1. Introduction

Breast cancer (BC) is one of the most common cancers worldwide and represents the leading cause of cancer-related death in women aged between 20 and 50 years [1]. Approximately, 75% of BC are estrogen-receptor-alpha (ER) positive. This marker, together with the progesterone receptor (PR) and human epidermal growth factor receptor 2 (HER2), currently guides the clinical management of BC patients through a tailored treatment approach. However, despite individualized treatment, recurrences still occur. Thus, there is a pressing interest in the comprehension of novel signaling markers that drive the different molecular pathways, which are relevant for normal mammary gland biology, BC development, progression, and response to treatment.

In this context, forkhead box A1 (FOXA1) is a forkhead-family transcription factor that prompted growing research interest due to its role in the hormonal-signaling network in normal breast epithelium and in BC.

FOXA1 is designated as a “pioneer factor” due to its ability to bind to highly compacted heterochromatin and expose genomic regions to other transcription factors. Actually, FOXA1 can adhere to the promotors of various genes associated with metabolic processes, the regulation of the signaling pathways, and the cell cycle [2,3].

Specifically, FOXA1 is essential for ER and AR attachment to chromatin and the subsequent transcriptional induction of luminal genes in BC cells, and, worthy of note, our group demonstrated that in luminal tumors, the expression of FOXA1 at the mRNA level is directly proportional to ER and AR levels [4]. In line with this, FOXA1 has been labeled as a “luminal gene”, promoting molecular, morphological, and clinical characteristics of luminal tumors regardless of their current intrinsic classification.

Concerning non-luminal BC, it was reported that in a subset of triple-negative tumors, namely luminal AR-positive tumors (LAR), FOXA1 induces AR function, resulting in an estrogen-induced-like proliferation, by directing AR to sites normally ER-occupied in luminal tumors [5,6,7,8].

In this paper, we provide a comprehensive review regarding the function and laboratory assessment of FOXA1, as well as of its prognostic and predictive role in different BC subtypes and in relation with ER and AR.

## 2. FOXA1: Principal Roles in Normal and Tumor Tissues

The FOXA subfamily of DNA-binding proteins (consisting of FOXA1, which is also known as HNF3*α* (hepatocyte nuclear factor *3*α), FOXA2 (HNF3β), and FOXA3 (HNF3γ)) was originally identified for its transcriptional regulation of the liver-specific genes transthyretin and alpha 1-antitrypsin [9]. These proteins bear an important role during embryonic development [10], cellular homeostasis, and steroid-hormone regulation [11]. Furthermore, FOXA proteins’ relevance in tumorigenesis and cancer progression has also been proven, establishing their role as significant biomarkers and possible targets of personalized-treatment approaches [12,13].

Specifically, the FOXA1 gene is located on chromosome 14q21.1 [14] and the transcribed region includes an intron spanning 5.300 bases only.

Beside the liver, the FOXA1 protein is also expressed in the breast, pancreas, prostate, bladder, colon, and lung, and is capable of binding promoters of more than a thousand genes that are involved in the regulation of intracellular-signal transduction and the cell cycle [15].

In particular, it has been demonstrated that the role of FOXA1 in different cancer locations may vary, e.g., from the tumor suppressor (for instance, in hepatocellular carcinomas by suppressing PIK3R1 expression [16]) or tumor promoter (for example, in breast and prostate cancer by binding to ERE and ARE motifs [17,18]), highlighting the complexity of its functions.

In the breast and prostate tissues, FOXA1 co-localizes with ER or AR in the nuclei, interacts with cis-regulatory regions in heterochromatin, and enhances the interaction of the hormone receptors with chromatin. Actually, FOXA1 acts as a “pioneer transcription factor” that associates with compact chromatin to increase its accessibility and facilitates the recruitment of other transcription factors, including nuclear receptors [19,20].

FOXA1 is indispensable for normal development of the breast [21,22], having a key role during mammary morphogenesis [21,23], being required for full ER activity [24], and directly interacting with GATA3 [21,24,25,26,27]. Ghosh and co-workers suggested that downregulation of FOXA1, following its hypermethylation in normal breast tissue, may contribute to the attenuation of ER function, impacting breast tumor development [28].

The function of FOXA1 in tumor development and progression is a matter of ongoing debate. It is well established that cancer-cell proliferation is impaired upon FOXA1 depletion [29] since ER and AR transcription is FOXA1-dependent both in breast and prostate cancer cells.

**Figure 1:**
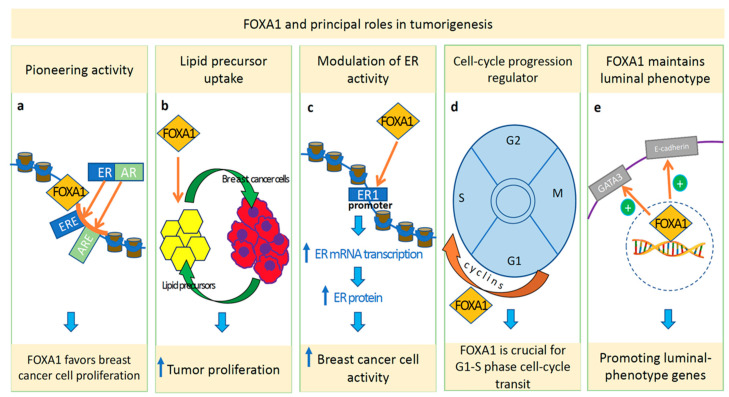
Graphical illustration of principal roles of FOXA1 in tumorigenesis that include pioneering activity (**a**), lipid-precursor uptake (**b**), modulation of estrogen-receptor (ER) activity (**c**), regulation of cell-cycle progression (**d**), and maintenance of expression of luminal-specific genes (**e**).

Specifically, due to its pioneering activity, FOXA1 binds to condensed chromatin, facilitating the subsequent attachment of estrogen/androgen and other linage-specific transcription factors [17,30] (Figure 1a).

Further mechanisms have been proposed to explain the role of FOXA1 during progression in different cancer models. In particular, FOXA1 is able to: (1) mediate the uptake of extracellular lipid precursors to increase tumor proliferation [29,31]; (2) modulate ER activity by binding to the ESR 1 promoter and favor both ER mRNA and protein expression in BC cells [24,30]; and (3) drive cell-cycle progression through the stimulation of cyclin D1 [32], cyclin E2, and E2F1 genes [18,33] (Figure 1b–d).

Moreover, during cancerogenesis, FOXA1 directly leads to a transcriptional increase in various downstream genes that specifically promote the luminal phenotype, such as the E-cadherin *CDH1* gene [15,30,34] and GATA3 [25,35] (Figure 1e), and repress basal differentiation [25,36].

A study performed on a mouse model by Sribenja and co-workers [37] reported that reduced FOXA1 expression alters luminal-cell differentiation, showing that its deletion in the mammary epithelium favors a two-fold increase in the portion of luminal-progenitor cells and reduces ER-positive cells. In line with these findings, a study by Badve and colleagues [38] performed on 404 patients with BC demonstrated that FOXA1 expression tested on tissue microarray correlates significantly with ER (*p* = 0.000001), PR (*p* = 0.00001), and luminal subtype A (*p* = 0.000001).

Worthy of note, FOXA1 mRNA and protein levels are also closely related with those of AR in hormone-dependent cancer models [4]. AR is expressed in a vast majority of ER-positive tumors and a limited fraction of TNBC, called LAR tumors [39]. In this setting of TNBC, several studies confirmed that high AR mRNA expression is strongly associated with high FOXA1 mRNA expression. In line with this, a study by Robinson and colleagues [8] performed on the ER−/AR+ MDA-MB-453 cell line revealed that the AR-binding profile is similar to that of ER in BC cells and that AR functionality depends on FOXA1 since its silencing inhibits AR-binding.

Furthermore, it has been reported that in apocrine TNBC, the ERE sites, which are normally occupied by ER in luminal tumors stimulating proliferation, are bound by AR under the guidance of FOXA1 [8]. In addition, Guiu et al. [40] suggested that TNBC with AR and FOXA1 co-expression may demonstrate luminal-like tumor behavior. Together, these data indicate that FOXA1 is able to maintain the BC luminal phenotype also through ER-independent mechanisms and directly repress the basal signature, stimulate apoptosis [36], and prevent the epithelial-to-mesenchymal transition [25,31,41,42].

Worth of note, overexpression of FOXA1 and HER2 has already been demonstrated to be strongly associated with ER-negative breast tumors [5]. In ER-/AR+BC, FOXA1 is also implicated in the HER2/ERBB2 pathway, playing a key role in cell proliferation and viability via the direct influence on ERK phosphorylation [5,42]. FOXA1 has been described as a transcription factor for HER2 [5,43] as well as a key regulator of HER2+ BC cell identity and adaptive reprogramming [44]. In detail, it has been proposed that FOXA1 is capable, via Junctional Adhesion Molecule-A (JAM-A) activation, of translocating to the nucleus, directly modulating HER2-gene transcription [44]. The shared cell lineage of HER2+ BC with other luminal BCs prompted deeper investigation into a potential role for FOXA1 in HER2+/ER− BC [5,45].

## 3. Evaluation of FOXA1 Expression: Immunohistochemistry and Gene Expression Analysis

Immunohistochemistry (IHC) and gene expression analysis are the most reported techniques for FOXA1 evaluation. However, interlaboratory reproducibility may be challenging and should be considered during data interpretation.

IHC analyses represent the most common assessment method but the obtained result may be influenced by the different types of used antibodies (monoclonal versus polyclonal), antibody origins (mouse/rabbit/goat), IHC protocols (dewaxing, hydration, antigen retrieval, antibody dilution, and incubation times), evaluation processes, specific cut-off values in terms of the percentages of nuclear staining, etc. In Table 1, we summarized data from previous studies (published since January 2016 until May 2021) performed on different BC models considering the clones, species of origin, dilutions, and manufacturers. Based on our personal experience, anti-FOXA1 mouse monoclonal antibody (2F83, Ventana-Roche), being prediluted and performed using a fully automated IHC staining system, is less prone to technical and analytic variability, and demonstrates a strong and reliable signal.

In a previous work from our group [4] that aimed to determine the specificity of FOXA1 antibody, we compared gene expression levels (using qPCR) with IHC results and found a strict correlation between FOXA1 mRNA and protein expression.

Worthy of note, FOXA1 is among the profiling genes in the PAM50 (Prosigna^®^) tumor subtyping test [63,64], simultaneously measuring the expression levels of 50 target genes plus eight house-keeping genes through a single hybridization reaction.

## 4. Prognostic and Predictive Roles of FOXA1

### 4.1. Luminal Breast Cancer

The prognostic and predictive impact of FOXA1 in BC remains a matter of debate. In general, a high level of FOXA1 expression has been associated to a better outcome in ER + BC. Although studies providing a mechanistic explanation of this clinical evidence are lacking, this association is probably due to a direct influence of FOXA1 on specific promoters (such as the p27 cyclin-dependent kinase inhibitor) that reduce ER-pathway activity [15]. A meta-analysis including nine studies comprised of 6386 patients affected by BC suggested that high FOXA1 expression positively influences disease-free survival (pooled HR: 0.43, 95% CI: 0.23–0.81; *p* < 0.05) and OS (pooled HR: 0.39, 95% CI: 0.26–0.60; *p* < 0.05) [65]. However, a sub-analysis including only the two studies considering ER + BC did not reveal a statistically significant association between the FOXA1 expression level and DFS or OS.

On the other hand, more recently, several works performed on ER + BC reported that FOXA1 expression was significantly associated with a favorable prognosis [4,47,66,67] also in tamoxifen-treated BC patients [68] and was a predictor of late recurrences [47]. Furthermore, it was demonstrated that FOXA1 expression in ER+ metastatic BC from various anatomical sites is strongly related to better overall survival (OS) and distant metastasis-free survival both in uni- and multivariate analyses [54,66,69].

In agreement with these data, FOXA1 is, in general, related to prognostically favorable characteristics, such as a low histological grade, a smaller tumor size, an absence of nodal metastasis, PR expression, HER2-negative status, and low levels of Ki67 [69].

In addition, data from our previous study [4] suggested that in the subset of ER + BC, there is an independent favorable prognostic value of FOXA1 that appears to be stronger than AR expression. Moreover, Ademuyiwa et al. suggested that FOXA1, negatively correlating with the recurrence score, is a more cost-effective pathological marker than the Oncotype DX multigene prognostic assay [70]. From a predictive point of view, the data regarding FOXA1 and the response to treatment in ER + BC are still debated and need a more comprehensive analysis based on large case series.

In particular, concerning endocrine treatment, FOXA1 has been reported both as a positive and negative marker of response. On the one hand, various studies reported that mutations within the FOXA1 promoter result in an endocrine-resistant cell growth and metastasization [17,71,72]. This is due to the ER-binding-landscape reprogramming triggered by FOXA1, resulting in an ER-activity increase that subsequently induces cellular tolerance to anti-ER treatment. On the other hand, a study by Tanaka et al. [62] suggested that despite no significant correlation occurring between the FOXA1 status and the efficacy of endocrine treatment, there was a notable decrease in the FOXA1 expression level in post-treatment samples. Similarly, it has been described that FOXA1 levels are decreased in pleural BC metastases after adjuvant endocrine therapy, a finding associated with poor outcome and endocrine-therapy resistance [56].

Data regarding the predictive role of FOXA1 and chemotherapy treatment in ER + BC are more unanimous, showing that high expression levels of this marker, inducing the luminal phenotype, are generally associated with poor response.

Kumar et al. suggested [73] that upon FOXA1 knockdown in luminal MCF-7 and T47D cells, there was an increase in the sensitivity towards chemotherapeutic agents, such as doxorubicin and paclitaxel. Comparable results were obtained by He et al. [74] that demonstrated a FOXA1-negative regulation of IFN signaling, inhibiting the immune response in ER + BC and promoting chemotherapy resistance. Clinical studies performed on ER + BC patients that underwent neoadjuvant chemotherapy treatment confirmed that high levels of FOXA1 result in a low rate of pathological complete response [75,76].

### 4.2. Non-Luminal Breast Cancer

Compared to ER + BCs, FOXA1 is expressed in a limited fraction of non-luminal tumors. However, it is always closely related to AR expression and molecular apocrine BC [57,77]. From a prognostic point of view, different from luminal BC, it has been shown that high levels of FOXA1 may lead to a less favorable outcome [52,57].

A paper from Mangia et al. [52] reported poor disease-free survival rates in AR+/FOXA1+ TNBCs compared to other TNBC tumors, identifying a specific subgroup of TNBC patients with poor prognosis and low levels of Tils and PD-L1. Similar data were stated in another study [57] performed on 333 non-metastatic TNBC, which found the AR+/FOXA1+ immunophenotype to be associated with significantly shorter recurrence-free and overall survivals.

Adverse outcome in FOXA1-positive TNBC could be justified by their low chemosensitivity, as reported in in vitro and in vivo studies. Worthy of note, in the MDA-MB-231 AR+ cell model, FOXA1 overexpression led to an increase in drug resistance and anchorage independence [73].

As discussed above, FOXA1 is able to maintain a less aggressive status, creating a “luminal” pattern in BC even due to independent ER mechanisms, inducing E-cadherin expression, and decreasing the cell migratory capacity [36,78]. These characteristics may justify the chemotherapy resistance observed in clinical studies, including the neoadjuvant setting; positive rates of FOXA1, associated with AR expression, tended to be lower in the pCR group (AR, 0 vs. 29%, *p* = 0.079; FOXA1, 8 vs. 29%, *p* = 0.233) among a series of patients receiving neoadjuvant chemotherapy [79]. Together, these data pave the way to the correct identification of a subgroup of TNBC patients in which chemotherapy may be ineffective and, thus, requires alternative treatments.

To date, the information regarding the prognostic and predictive role of FOXA1 in HER2-positive BC are limited. Very recently, in a study by Cruz et al. [44], the authors revealed that coincident high-mean mRNA expression of JAM-A, HER2, and FOXA1 is associated with poorer survival outcomes in HER2-positive (but not HER2-negative) patients with either breast or gastric tumors. Moreover, one of the possible explanations suggested regarding the acquired resistance to HER2-targeted therapies in BC patients was a link between JAM-A, β-catenin, and FOXA1 that triggers HER2-independent tumor proliferation via HER3 activation [80].

## 5. Conclusions

Significant progress has been made in understanding the biological role of FOXA1 in BC development, differentiation, and progression. These complex data indicate the need to perform additional research addressing the potential opportunity to target FOXA1 using specifically tailored treatment approaches. To date, FOXA1 expression, assessed by IHC, demonstrates a potential prognostic role in both luminal and non-luminal BC.

In our opinion, from a practical point of view, the high expression of FOXA1 in ER + BC may allow for predicting a good prognostic outcome, potentially supporting the clinicians’ decision to omit chemotherapy (also as a neoadjuvant approach) in luminal BC. On the other hand, in ER cases, FOXA1 may be useful in identifying patients that will not respond to chemotherapy, allowing for identifying a subgroup of TNBC that is not suitable for neoadjuvant treatment. Hence, the routine assessment of FOXA1 may represent a valuable and instructive tool to refine BC patients’ prognosis.

Finally, since FOXA1 can be reliably assessed by IHC, it may represent a low-cost marker easily applicable to formalin-fixed and paraffin-embedded tissue during the routine diagnostic work-up. Further research in large prospective cohorts is warranted to validate the accuracy of these hypotheses.

## Figures and Tables

**Table 1:** Literature data regarding immunohistochemical analyses of FOXA1 expression.

Study	Clone	Species	Dilution	Manufacturer
Rangel et al. [4]	Monoclonal, 2F83	Mouse	prediluted	Ventana-Roche
Chen et al. [46]	Monoclonal, EPR10881, ab170933	Rabbit	1:100	Abcam
Horimoto et al. [47]	Polyclonal, ab23738	Rabbit	NR *	Abcam
Zhang et al. [48]	Monoclonal	Rabbit	1:100	Bioss, China
Byun et al. [49]	Polyclonal, ab23738	Rabbit	1:10,000	Abcam
Cheng et al. [50]	Monoclonal, sc-101058, Q6	Mouse	NR	Santa Cruz Biotechnology
Nelson et al. [51]	Monoclonal, ab173287	Rabbit	1:4000	Abcam
Mangia et al. [52]	Monoclonal, 2F83	Mouse	1:200	Merck Millipore
Dai et al. [53]	NR	NR	1:100	Abcam
De Lara et al. [54]	Monoclonal, 2F83	Mouse	1:100	CELL MARQUE
Kutasovic et al. [55]	Monoclonal, 2F83, ab40868	Mouse	1:100	Abcam
Schrijver et al. [56]	Monoclonal, WMAB-2F83	Mouse	1: 100,000	Seven Hills Bioreagents
Guiu et al. [57]	Polyclonal, HNF-3α/β (C-20)	Goat	NR	Santa Cruz
Mai et al. [58]	Monoclonal	Mouse	1:50	Sigma
Mori et al. [59]	Monoclonal, EPR10881	Rabbit	NR	Abcam
Humphries et al. [60]	Monoclonal, ab55718	Mouse	1:500	Abcam
Davis et al. [61]	Monoclonal, 2F83	Mouse	1:100	Millipore
Tanaka et al. [62]	Monoclonal	Mouse	1:500	Abcam

* NR: not reported.

## Data Availability

Not applicable.
